# Tidal Records as Liquid Climate Archives for Large-Scale Interior Mediterranean Variability

**DOI:** 10.1038/s41598-018-30930-8

**Published:** 2018-08-22

**Authors:** Angelo Rubino, Davide Zanchettin, Alexey Androsov, Naum E. Voltzinger

**Affiliations:** 1 University Ca’Foscari of Venice, Department of Environmental Sciences, Informatics and Statistics, Via Torino 155, 30172 Mestre, Italy; 2 Alfred Wegener Institute Helmholtz Centre for Polar and Marine Research, Postfach 12-01-61, 27515 Bremerhaven, Germany; 3 The St. Petersburg Department of P.P. Shirshov Institute of Oceanology, RAS, 30, Pervaya Liniya, 199053 St. Petersburg, Russia

## Abstract

Characterization of interior ocean variability is necessary for understanding climate. Water mass evolution shapes ocean-atmosphere interactions and contributes to determine timescales for global and regional climate variability. However, a robust assessment of past state and variability of the ocean interior is prevented by sparseness/shortness of historical subsurface observations and uncertainties affecting proxy-based reconstructions. Here, we propose a novel approach to infer past large-scale interior ocean variability with unprecedented accuracy and temporal resolution. It exploits links between stratification determined by “large-scale” water mass distributions and local dynamics. We characterize interannual interior ocean variability in the Mediterranean Sea in the early 20th century contained in tidal measurements in the Strait of Messina, and demonstrate the general applicability of our method, paving the way to a new approach to analyze historical oceanographic records: Regions where different water masses are known to collide can thus act as magnifying glasses for basin-scale interior ocean variability, hence providing “liquid archives” for climatology.

## Introduction

Among the intricate interactions involved in the oceanic (eco)system functioning and variability, a crucial role is ascribed to phenomena connecting surface, interior and deep ocean. It is therefore obvious that a profound understanding of their dynamics and variability is pivotal to our understanding of climate evolution. However, the strongest limit to progress in our comprehension of the ocean (eco)system is given by a tremendous lack of observations, particularly in the deep and abyssal layers^1^. The relevance and urgency of global oceanographic observing systems is testified by the major measurement programs launched and/or announced in recent years (see, e.g., http://www.o-snap.org, and MedSHIP^2^). That for the future, but what about past variability of the interior ocean? Our current knowledge encompasses just a small fraction of climatically-relevant episodes of ocean variability that occurred in the past few centuries.

In the Mediterranean Sea (see Supplementary Fig. S1), for instance, large changes affecting the interior water masses have been observed in recent years^3–6^. In particular, the Eastern Mediterranean Transient (EMT) was a major climate shift that occurred at the end of the eighties and culminated in the nineties^7^: Abyssal waters of Aegean origin replaced Adriatic deep waters (AdDW) in the bottom layers of the Ionian basin^3,8^. This phenomenon triggered large transformations in the interior and deep dynamics of the Eastern as well as the Western Mediterranean basin, which are still ongoing^9,10^. The EMT is considered as the most significant intermediate-to-deep Mediterranean overturning perturbation observed in the instrumental period^7,10^. Its preconditioning involves, among other factors, paths of mere interior water-mass variability, like the one linked to alternating upper-ocean vorticity in the northern Ionian basin and recently baptized as Bimodal Oscillatory System, or BiOS^11,12^. It has been debated whether EMT-like episodes can be regarded as recurring events^6^. A recent reconstruction based on the analysis of sediments in the Mediterranean basin supports the hypothesis that they occurred under particularly favorable conditions also in the preindustrial past^13^. However, accurate dating of these episodes of interior ocean variability remains a grand challenge of current oceanography: High-resolution observations and/or reconstructions of the interior ocean - a sort of a “liquid climatic archive” - are missing.

Among the oldest sources of direct information about ocean state and variability are tide-gauge records: For instance, the oldest tidal measurements available along the Eastern Mediterranean coasts extend back to the late 19th century^14,15^. Besides the tides, for whose determination they were originally conceived, these records preserve a trace of many local atmospheric and oceanic phenomena, like, e.g., wind-induced sea-level variations, inverse barometer effect, and, particularly, modes of large-scale climate variability^16^. The latter, obviously, include those arising from interior ocean dynamics, such as phenomena related to interannual-to-decadal variability of seawater density.

Here, for the first time, we use historical tide-gauge observations and numerical simulations of tidally-dominated sea strait dynamics to demonstrate that tide-gauge data can reveal information about the long-term interior ocean variability. As a result, amplitude and phasing of interior ocean variability during historical periods for which direct measurements of the water column stratification do not exist can be inferred with a temporal resolution and a level of accuracy not achievable by other existing measurements or traditional proxies. Past studies following a similar approach focused on the short-term dynamics arising through the interaction of sea-level gradients and ocean stratification (see, e.g., refs^17–21^). Brandt *et al*.^22^ recognized the relevance of larger-scale oceanic features on the undular tidally-induced dynamics in the Strait of Messina, but they did not focus on the long-lasting behavior of the interior ocean variability under the effect of tides. Our contribution is intended as a starting point for filling this gap of knowledge.

## Results

**Figure 1:**
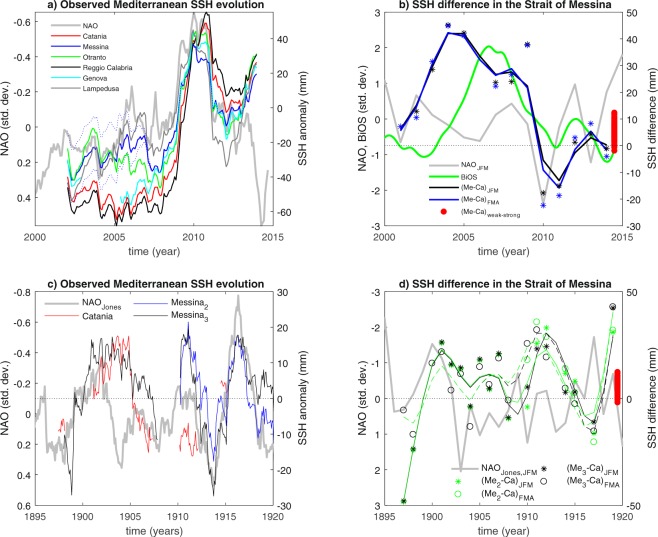
Observed SSH evolution and its relation with modes of large-scale variability. (**a**) Smoothed (25-point running average) monthly mean anomalies of SSH along the Italian coast of Fig. 2a, plus Genova in the Ligurian Sea, and of the deseasonalized monthly NAO index, for the period 2000–2015. (**b**) Winter (JFM: Jan. to Mar.; FMA: Feb. to Apr.) average SSH difference between Messina and Catania (stars: original seasonal-average data; continuous lines: 5th-order polynomial fit to the data) and winter (JFM) NAO and BiOS indices. The BiOS index is standardized and smoothed with a 13-month running average, for the period 2000–2015. (**c**) Smoothed (25-point running average) monthly mean anomalies of SSH in Messina and Catania, and of the deseasonalized monthly NAO index, for the period 1895–1920. (**d**) Winter (JFM and FMA) average SSH difference between Messina and Catania (stars: original seasonal-average data; continuous lines: model fit to the data) and winter (JFM) NAO indices, for the period 1895–1920. In panels a and b SSH anomalies are with respect to the smoothed 2007–2015 climatology. For Messina, in panel a, the continuous and the dashed lines indicate the ensemble mean and envelope, respectively, that account for the discontinuity in the data (see methods). In panels c and d, subscripts 2 and 3 indicate that two polynomial trends - 2nd and 3rd order - are removed from the Messina SSH data after the Messina earthquake. Fitting is with a 5th-order polynomial model in b and with a smoothing spline in d. The red dots in panels b and d show the range of differences between Messina and Catania SSH simulated by the model.

**Figure 2:**
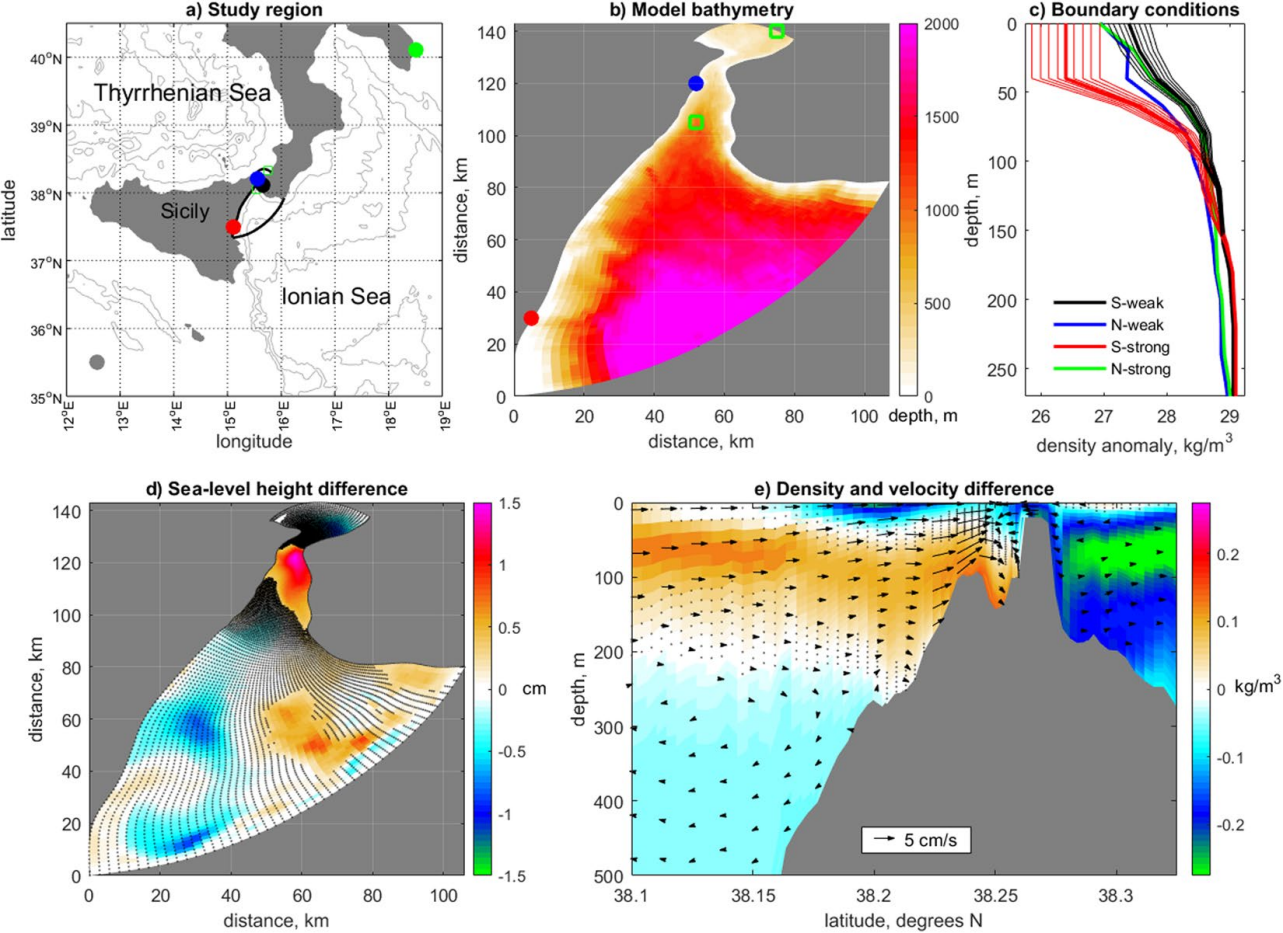
(**a**) Study region with location of the tide gauges shown in Fig. 1a indicated with circles (same color code) and model domain (thick black contour). (**b**) Bathymetry of the Strait of Messina with the approximate location of the Messina station (blue circle), the Catania station (red circle) and the CTD stations where the density profiles illustrated in panel **c** were measured. (**c**) Density profiles used in the two experiments as boundary conditions. For the southern boundary conditions the thick lines indicate the observed data, while the thin lines indicate the scaled profiles. (**d**) SSH difference between ensemble averages of SSH values calculated during the last tidal period in the strong and weak stratification experiments (weak minus strong). Dots indicate grid points where the difference is statistically not significant. (**e**) Difference between ensemble averages of seawater density (shading) and velocity (arrows) calculated during the last tidal period in the strong and weak stratification experiments (weak minus strong). Representation for density follows representation for SSH in panel d. A subset of the velocity data is shown (every three gridpoints horizontal, and every 10, 5 and 3 gridpoints for depth below 100 m, 250 m and bottom, respectively). Only significant differences in the velocities are shown.

It is known that interannual-to-decadal evolution of sea-surface height (SSH) along the coasts of the Mediterranean basin largely reflects the imprint of atmospheric circulation changes related to the North Atlantic Oscillation, or NAO^15,23^. Figure 1a illustrates such connection between the NAO index and SSH variability along the Italian coast: At all tide gauges, sea level coherently responds to the NAO forcing, and particularly to the strong negative NAO event in 2010. However, SSHs also express interannual variability, which cannot be explained by the NAO alone, as exemplified in recent years by the divergent evolutions of NAO and SSHs (Fig. 1a). Such discrepancies account for the complexity of externally-driven and interior ocean dynamics contributing to SSH variability. Focusing particularly on wintertime, when the NAO is dominant and ocean dynamics is less affected by the presence of a near-surface seasonal thermocline, substantial differences characterize the SSH evolution measured in the stations of Messina, located within the tidally-dominated Strait of Messina, and Catania, located along the eastern coast of Sicily, less than 100 km apart from Messina (Figs 1b and 2a). The SSH difference shows prominent variations (~2 cm), including a slow transition between around 2005 and 2010 from higher to lower values in Messina relative to Catania. Besides the concomitant peaks in 2010, the SSH difference between the two stations does not appear to be linked to the NAO; instead, it rather well follows the BiOS evolution (Fig. 1b). Therefore, the data reveal portions of interannual-to-decadal variability apparently related to interior oceanic processes.

To explain the origin of these relationships emerging from the observed data, we carried out simulations using a very high resolution, hydrostatic numerical model for the description of tidally-induced dynamics in stratified sea straits on an *f*-plane (Fig. 2a,b). The use of this model, which has demonstrated to reproduce accurately the hydrodynamics of the strait of Messina^24^, is necessary, due to the paucity of observational information in the area. Two experiments were designed to describe the effects of different stratifications at the northern and southern boundaries of the Strait of Messina (Fig. 2c)^22^. Specifically, the mean SSH responses to the following boundary conditions were investigated: First, stratifications resembling situations when an excess of Modified Atlantic Water, or MAW, occupies the near-surface layers of the southern approaches to the strait (ensemble “strong stratification”), as occurred, e.g., around 2005; then, stratifications resembling situations occurring when the density is higher south rather than north of the strait throughout the water column (ensemble “weak stratification”), as observed, e.g., around 2010. These two commonly encountered stratifications are known to occur during anticyclonic and cyclonic phases of the BiOS, respectively. We note, however, that both conditions cannot be ascribed univocally to the BiOS alone. Ten-member ensembles are considered to account for uncertainty in the stratification at the southern boundary. The red dots in Fig. 1b depict the range of SSH differences between Messina and Catania as simulated by our numerical model. The model captures part of the observed variability. Unavoidably, it cannot reproduce the full range of observed values. This is very likely because observed extreme variations are associated with very localized “jet-like” dynamics which can be simulated only approximately^21^, due particularly to the approximations induced by initial and boundary conditions.

In the simulations, the difference between conditions of strong and weak stratification (weak minus strong) at the southern boundary results in a significant positive SSH anomaly within the strait relatively to the open sea (Fig. 2d). This differences, in a sea strait characterized by a very complex bathymetry, is due to the different dynamics induced by the tide in the presence of a variable stratification and under the effect of the Coriolis force. In particular, the difference yields significant northward velocity anomalies in the surface and intermediate layers of the Ionian basin as well as in the subsurface layers of the Tyrrhenian sea (Fig. 2e) that can be explained as follows: An excess of MAW in the southern approaches to the strait (experiment with “strong stratification”) induces on both sides of the strait near-surface velocities that result in an almost vanishing average surface flow and in a strong intermediate southward flow in the Ionian Sea with a core at about 100 m depth. When, instead, the seawater density is higher south than north of the strait throughout the water column (experiment with “weak stratification”) surface northward tidal currents are stronger than surface southward tidal currents (which produces an average northward flow), while, with exception for the near-sill area, intermediate tidal currents in the Ionian Sea are of comparable magnitude. As a result of such a complex average tidal flow distribution in the two cases (weak minus strong), we observe a noticeable average northward tidal flow in the upper layer of the Ionian basin (Fig. 2e). In the sill area the situation is rendered even more complex, due to the presence, in both cases on different time intervals within the tidal cycles, of regions characterized by supercritical flow conditions. As a result, in the weak minus strong anomalies a convergence region is found around the strait’s sill which is responsible for positive SSH anomalies there (Fig. 2d).

**Figure 3:**
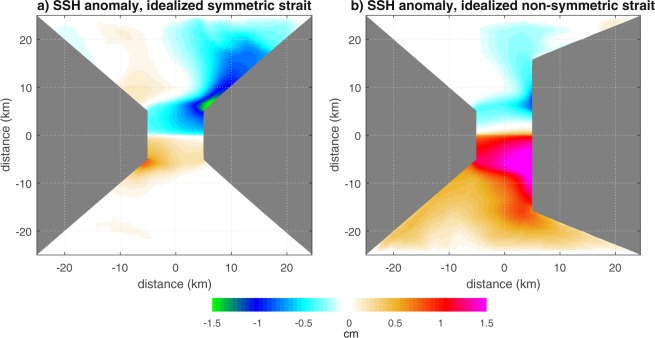
Simulated sea surface height anomalies for the case of (**a**) symmetric and (**b**) asymmetric idealized strait. The vertical density profiles imposed as boundary conditions are those illustrated as thick lines in Fig. 2c.

The development of strong near-surface velocities under strong stratification is consistent with the strong near-surface jets observed to propagate northward under similar conditions. The presence of an intermediate jet in the Tyrrhenian sea under weak stratification is consistent with known dynamics in the Strait of Messina^21,25^. The strength and position of these jets contribute to determine, inside the strait, significant differences between SSH under strong and weak stratifications. But, beyond the observed characteristics typical of the Strait of Messina, these findings seem to express a property common to sea straits and, more generally, to regions where different water masses usually collide. Well-defined, persistent spatial features characterize the anomalous patterns of mean SSH obtained by applying the same boundary conditions as in the realistic case to two idealized situations: a symmetric and an asymmetric tidally-dominated strait (Fig. 3). The signs of the anomalies in the approaches to the strait’s sill are, for both bathymetries, qualitatively consistent with those simulated for the realistic case, indicating its robust dependency on the density fields imposed at the boundary. We note that, as for the realistic case, the asymmetry emerging in the simulated SSH anomalies is a result of the combined action of the baroclinic density gradient due to the imposed stratification as well as of the deflection induced by the (although relatively small) Coriolis force. This highlights how the bathymetry of the strait can substantially influence the details of the spatial SSH response pattern to the imposed oceanic forcing, hence rendering it distinctive for each strait.

Thus, the SSH anomaly gradient between the strait interior and the open sea reflects seawater density anomalies at the strait boundaries. For the Strait of Messina, the latter reveals the local imprint of large-scale interior oceanic features, among them the phase - cyclonic or anticyclonic - of the BiOS. Thus, Fig. 1b discloses a real dynamical property linking interior large-scale and local sea surface variability.

Based on these observational and simulated evidences, we investigate the difference between historical SSH measurements at the gauges of Messina and Catania available for the early 20th century (Fig. 1c) to infer past variations in the internally-induced large-scale circulation of the Ionian Sea. The record of measured SSH differences features prominent interannual-to-decadal variability, especially in wintertime, which is not directly linked to NAO atmospheric forcing (Fig. 1d). We posit that such SSH differences trace interior ocean variability in the Ionian Sea similarly to what was observed in the last two decades (Fig. 1b) and simulated numerically (Fig. 2). Accordingly, we use tidal records to infer past interior ocean variability. The most prominent feature of the observed anomalies is a persistent decadal oscillation, whose positive phases are found around 1900, 1911-12 and 1919 CE, and whose negative phases are found around 1896, 1905 and 1916–17 CE (Fig. 1d). These oscillations could reflect either prominent episodes of BiOS variability or a larger mass redistribution in the area, as the one observed during the EMT. The latter conjecture is corroborated by a recent paleoceanographic record from the central Mediterranean that identifies recurrent signals of paleo-EMTs during the past centuries, including an event in 1910 ± 12 years^13^. Our inference is consistent with the hypothesis that an EMT-like event occurred in the early 20th century. The higher temporal resolution of our record significantly improves the temporal characterization of the event, and allows to recognize that, possibly, a series of separate episodes contributed to its manifestation.

## Discussion

In this study, for the first time, we illustrate the possibility of inferring aspects of large-scale interior ocean variability in the central Mediterranean basin from local tidal observations in the tidally-dominated area of the Strait of Messina. Observational and simulated evidences have been proposed to support the viability of our approach. In the Ionian Sea, two phenomena can yield a straightforward dynamical interpretation of the obtained results: On the one hand, the BiOS mechanism has been recently identified as a major mode of interannual-to-decadal variability of the interior ocean dynamics in the area and, on the other hand, an abrupt shift in the location of dominant deep-reaching convection seems to occur sporadically, the most recent of such events being baptized as the EMT. In this sense, our study also sheds light on the results by^24^ relating tidal phenomena within the Strait of Messina to the decadal evolution of large-scale features associated to interior ocean dynamics and demonstrates how their complex variability is influenced, among other things, by pre-existing water-masses and bathymetric constrictions.

Analogous phenomena of the interior ocean affecting the long-term density structure of the water column must be acting elsewhere in the World Ocean, where different dynamically active water masses are known to collide. Hence, coherent long-lasting differences between past SSH from tidal records of neighboring stations could yield valuable information to reconstruct such dynamics: They would represent a “liquid archive” for the mostly unknown - till now irremediably lost - interannual-to-decadal variability of the interior ocean during periods of the past for which direct experimental measurements do not exist. To achieve this goal, one should compute SSH differences between neighboring tidal records and seek for robust correlations with density stratifications expressing observed modes of large-scale ocean variability. The use of a numerical model to simulate the site’s oceanography can help to identify the underlying linking mechanism.

**Figure 4:**
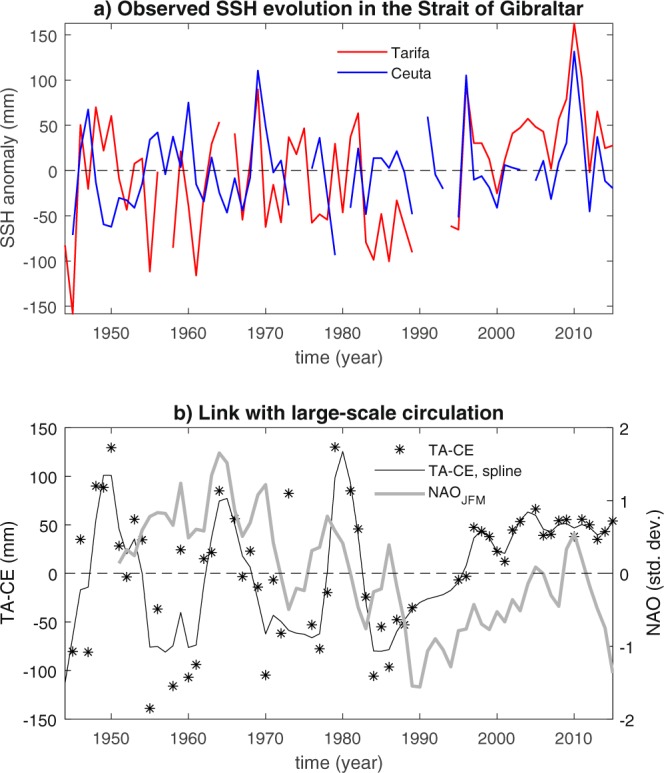
Attempt to infer oceanic variability in the Mediterranean Sea during the last six decades using tide-gauge data from Tarifa (TA) and Ceuta (CE) in the Strait of Gibraltar. (**a**) Observed winter (JFM) CE and TA SSH anomalies with respect to the 1950–2015 period. (**b**) Comparison between observed difference between winter SSH measurements in Tarifa and Ceuta (black stars: original data; black line: associated spline smoothing), and the smoothed winter NAO index (gray, 3-year running average).

Figure 4, for instance, illustrates the evolution of the winter SSH signals observed in the gauges of Tarifa and Ceuta, in the Strait of Gibraltar (see Supplementary Fig. S1), and their difference. The time series of the differences between the two records displays a prolonged negative phase in the mid and late 1980s, i.e., during the same period of the EMT. This event marks a transition from a period dominated by strong near-decadal variability, with peak positive values around 1950, 1964 and 1980, to a period dominated by a prolonged positive anomaly with weaker interannual-to-decadal variability superimposed. The existence of a causal link between the observed EMT and the changes in tidal characteristics within the Strait of Gibraltar remains unavoidably speculative. Nonetheless, our results add further evidence to the picture identifying the late 1980s as a transition period characterized by strong and still poorly understood oceanic variability in the Mediterranean Sea. Future studies exploring more quantitatively the link between tidal dynamics in the Strait of Gibraltar and the EMT will certainly contribute to advance understanding of the mechanism linking more intense convective activity in the Eastern Mediterranean basin and enhanced MAW transport throughout the Western Mediterranean basin^13^. We note that the NAO evolution dominates tidal variability during certain periods, e.g., in the 1970s and in the most recent decades (Fig. 4b): This is again expected due to atmospheric forcing of tidal dynamics. Arguably, the contribution of interior ocean dynamics to the observed SSH variability can thus be better identified focusing on periods characterized by a marked disagreement between NAO and the SSH difference, like, for instance, the mid and late 50s and the mid-80s. Another aspect to account for is that NAO, as well as other large-scale modes of atmospheric circulation, can significantly imprint on the thermohaline properties in key areas of the Mediterranean Sea through a variety of mechanisms^26^, hence indirectly interfering with interior ocean variability.

In conclusion, our novel approach allows to identify accurately dated, seasonal events of past interior ocean variability. The identification of tidal measurements within sea straits (or, more generally within sea regions where different water masses are known to collide) as a “liquid climatic archive” contributes to face, in an innovative way, one of the grand challenges of modern oceanography.

### Data

Monthly-mean relative sea-level height data used in Fig. 1a,b are revised local reference data obtained from tide gauges and provided by the Permanent Service for Mean Sea Level (PSMSL^27^). The Catania station is Catania 2 (station ID: 2094). For the Messina station, metric data are used (Messina 2, station ID: 2081) for which a discontinuity in the data is reported as likely to have occurred in April/May 2006. We homogenize the pre- and post-shift time series by accounting for uncertainties linked to the calculation of pre- and post-shift averages over different periods. The shift is assumed to have occurred in May 2006. To this purpose, an ensemble of revised time series for Messina is generated, that account for different possible corrections for the discontinuity. Specifically, the revised time series are obtained by removing the difference between each of the pre-shift averages calculated for periods covering from between January 2001 and May 2005 to May 2006, with the initial time increased progressively by one month, and each of post-shift averages calculated for periods covering from between June 2006 and June 2007 to May 2010, with the final time increased progressively by one month. We thus obtain 1909 revised time series in the ensemble. The average of such ensemble is used in the calculation of SSH difference between Messina and Catania. The missing value in Messina 2 for January 2006 is filled by averaging the data for December 2005 and February 2006. We do not consider data from 2006 in the calculation of the tidal difference. Supplementary Fig. S2 illustrates the various phases of the pre-processing as well as all original relative sea-level data used in Fig. 1.

Monthly-mean relative sea-level height data used in Fig. 1c,d are metric data obtained from tide gauges and provided by PSMSL (Messina station ID 115; Catania station ID: 102). The data from Messina covering the early 20th century are homogenized by correcting for the increasing nonlinear trend that affects the measurements after the 1908 earthquake^15^. Specifically, to account for uncertainty in the trend, data from January 1909 onward are cleaned of their second-order and third-order polynomial components. Supplementary Fig. S3 illustrates the various phases of the pre-processing as well as all original relative sea-level data used in Fig. 1. The data used in Fig. 1c,d cover the periods 1896–1971 with long periods of missing data (particularly there is a gap between 1920 and 1960) for Catania, and 1897–1923 for Messina.

The BIOS index is the updated index originally published by ref.^28^. The index is defined as the 13-month smoothed average time series of the zonal component of the surface geostrophic currents computed for the northern portion of the Ionian Sea.

More specifically, following^12^, negative BiOS values correspond to the case when the upper layer of the Ionian Sea circulates cyclonically. In such a case, saltier Levantine Intermediate Water enters the Adriatic, which yields an increased probability of Deep Water Formation (DWF) and, consequently, of denser Adriatic Deep Water (ADW). This newly formed ADW, filling the bottom layers of the Ionian basin, induces a stretching of vorticity and hence a reversal of the overlaying circulation, which becomes then anticyclonic. This forces MAW to enter the Adriatic, which leads to a decreased probability of DWF and, consequently, to lighter ADW. As a result, this newly formed ADW leads to a squeezing of vorticity which reverses the circulation again.

The NAO index used for Fig. 1a,b is the monthly NAO index provided by the Climate Prediction Center (CPC) of the National Weather Service of the NOAA. The index is obtained as the first principal component from a rotated principal component analysis performed on monthly mean standardized 500-mb isobar height anomaly data in the Northern Hemisphere. Details of the procedure are available on the NOAA-CPC website at the http://www.cpc.ncep.noaa.gov/data/teledoc/telepatcalc.shtml.

The NAO index data used for Figs. 1c,d and 4b are from the index defined as the difference between the normalised sea-level pressure over Gibraltar and the normalised sea level pressure over Southwest Iceland^29^. This definition allows to use early instrumental data to extend this index back to 1823.

The vertical density profiles used in the model experiments are based on the experimental observations illustrated in^22^. Ensembles of profiles for conditions of strong and weak stratification at the southern boundary are generated by scaling the original profiles in^22^ by factors sampled in the range [0.8 1.2] at intervals of 0.05.

### Numerical model

The dynamics of tidally-dominated sea straits is simulated by solving a 3-D boundary-value problem for the equations of momentum, continuity, temperature and salinity, and turbulence characteristics in a realistic domain. The spatial frame is made by curvilinear boundary-fitted horizontal coordinates (the horizontal grid step spans from 50 m to 600 m, while the time step is selected to be 60 s in order to ensure numerical stability) and vertical σ-coordinate. The numerical method is composed of different split operators, particularly designed to obtain an accurate representation of advective processes and to retain the complexity of the solution’s vertical structure^24,30^. The model is described in detail in the Supplementary material, while^23^ provides a detailed evaluation of the model based on comparison with observational data. Hence the model includes the contributions to the dynamics brought by gradients of elevation and of density in a non-stationary, nonlinear, turbulent context, in the presence of realistic bathymetry for a rotating Earth.

For each simulation, 41 tidal periods are simulated, and the data from the last tidal period are retained for the statistical analysis.

The range of simulated SSH differences between Messina and Catania in Fig. 1b,d is obtained by first calculating the SSH difference in each realization in both the strong and weak experiments, then calculating the differences between all such estimates, independently of the experiment group.

### Statistical significance

The Wilcoxon rank sum test is used to calculate statistical significance at the gridpoint level. The 0.05 significance level is our reference to reject the null hypothesis of equal medians between the two ensembles, namely the strong and weak stratification ensembles.

## Electronic supplementary material

Supplementary Information
