# Supplementary material for: Tidal Records as Liquid Climate Archives for Large-Scale Interior Mediterranean Variability
Source: Sci Rep. 2018 Aug 22;8:12586. doi: 10.1038/s41598-018-30930-8 (PMC6105714; doi:10.1038/s41598-018-30930-8)
Supplement: Supplementary file 1 — Supplementary Information [file 41598_2018_30930_MOESM1_ESM.doc]

**Tidal Records as Liquid Climate Archives for Large-Scale Interior Mediterranean Variability**

Angelo Rubino, Davide Zanchettin, Alexey Androsov, Naum E. Voltzinger

**Supplementary Information**

**Detailed description of the numerical model**

Suppose that an undisturbed water surface coincides with the horizontal plane$X0Y$of a right-handed Cartesian coordinate system and the $0Z$- axis is directed upward. In such frame, let $Q$ be the initial strait volume. As time elapses, the volume will experience variations:$\hat{Q}=Q\times\left[0,\text{T}\right]$ depending on the free air-sea surface *ζ*, the sea bottom *h*, and the side surface $\partial Q, Q=$ $\left\{x,y,z; x,y\in\Omega, -h\left(x,y\right)\leq z<\zeta\left(x,y,t\right)\right\}, 0\leq t\leq\text{T}.$ Note that, in the previous expression, $\Omega\left(x,y\right)$ is the two-dimensional, horizontal domain. In $\hat{Q}$, for each time, we wish to determine the solution as a vector $\mathbf{q}=\left(u,v,w,\zeta,\rho\right),$ where $\mathbf{v}=\left(u,v\right)$ is the horizontal velocity, $w$ the vertical velocity, and $\rho$= $\rho$ (T,S,P) is the seawater density.

We introduce a $\sigma$- coordinate in the vertical and horizontal curvilinear coordinates (*ξ ,η)* fitted to the shape of $\Omega\left(x,y\right)$. Consider the transformation

$\xi=\xi\left(x,y\right) , \eta=\eta\left(x,y\right) , \sigma=\frac{z-\zeta}{H} , \tau=t ,$ (1)

with $H=h+\zeta, -1\leq\sigma\leq0 ,$ and the Jacobian $J=J_{*}H$, where $J_{*}=\partial\left(x,y\right)/\partial\left(\xi,\eta\right)$ is a planar Jacobian such that $0\neq J_{*}<\infty$. With an appropriate choice of four pairwise opposite segments of $\Omega$, $Q$ is mapped into a parallelepiped $Q_{*}$.

In the hydrostatic approximation, the pressure is then represented as

(2)

where $\rho_{0}$ is the mean value of the density, while$\rho'\left(\zeta,\eta,\sigma;t\right)$ is the deviation of *ρ* from its hydrostatic value. In the new variables the momentum, continuity, and the constituents of the density equations take the form of conservative contravariant fluxes (Androsov and Voltzinger, 2005):

$$\mathbf{v}_{t}+U^{i}\mathbf{v}_{\xi^{i}}+g\left(\zeta_{\xi}\nabla\xi+\zeta_{\eta}\nabla\eta\right)+g\rho_{0}^{-1}I_{\xi^{i}}\nabla\xi^{i}+f \mathbf{k}\times\mathbf{v}=$$

$=H^{-2}\left(\nu\mathbf{v}_{\sigma}\right)_{\sigma}+J^{-1}\left(KJg^{ik}\mathbf{v}_{\xi^{k}}\right)_{\xi^{i}} ,$ (3)

$J_{t}+\left(JU^{i}\right)_{\xi^{i}}=0 ,$ (4)

$\begin{matrix} S_{t}+U^{i}S_{\xi^{i}}=H^{-2}\left(\nu_{\rho}S_{\sigma}\right)_{\sigma}+\left(K_{\rho}Jg^{ik}S_{\xi^{k}}\right)_{\xi^{i}} \\ T_{t}+U^{i}T_{\xi^{i}}=H^{-2}\left(\nu_{\rho}T_{\sigma}\right)_{\sigma}+\left(K_{\rho}Jg^{ik}T_{\xi^{k}}\right)_{\xi^{i}} \end{matrix}\text{ }$ (5)

where $i,k=1,2,3$ and summation over repeated indices is assumed .The $U^{i}$ are the contravariant velocity components: $U^{1}=U=\mathbf{v}\cdot\nabla\xi,$ $U^{2}=V=\mathbf{v}\cdot\nabla\eta,$ $U^{3}=W=\sigma_{t}+\mathbf{v}\cdot\nabla\sigma+w\sigma_{z} ;$ $\xi^{1}=\xi, \xi^{2}=\eta, \xi^{3}=\sigma; \nabla=\left(\partial/\partial x,\partial/\partial y\right)$ where $f$ is the Coriolis parameter; $\mathbf{k}$ is the vertical unit vector; $\nu$ and $\nu_{\rho}$ are eddy viscosities; $K$ and $K_{\rho}$ are eddy diffusivities; and $g^{ik}=\nabla\xi^{i}\nabla\xi^{k}$ are the components of the contravariant metric tensor. The seawater density *ρ* is here determined by temperature and salinity, using the UNESCO formulas.

The sea level is computed by using the vertically averaged continuity equation.

Since $\left.W\right|_{\sigma=-1}=\left.W\right|_{\sigma=0}=0 ,$ this equation finally is

(6)

where and $\bar{\mathbf{v}}=\int_{-1}^{0} \mathbf{v} d\sigma.$

Let us now specify the boundary conditions. On the impermeable faces we have

$\left.U^{i}\right|_{\partial\Omega_{1}^{*}}=0.$ (7)

At the open boundary, the conditions are set in accordance with Androsov et al. (1995). In the above formulation of the problem, it is assumed that

$\left.\zeta\right|_{\partial\Omega_{2}^{*}}=\zeta_{\Gamma}\left(\tau\right) .$ (8)

The boundary conditions in the vertical is

$\begin{matrix} \left.JU^{i}\right|_{\sigma=-1+\varepsilon}=0 , \left.\nu JU_{\sigma}^{i}\right|_{\sigma=0}=0 ,\text{ } \\ \end{matrix}$ (9)

where $\varepsilon$ is the roughness length of the sea bottom. From (5) at the impermeable boundaries we have

$\left.\partial S/\partial n\right|_{\partial\Omega_{1}^{*}}=0 , \left.\partial T/\partial n\right|_{\partial\Omega_{1}^{*}}=0 .$ (10)

At the open sides, the characteristics of inflowing water masses are specified as $\left.S\right|_{\partial\Omega_{2}^{*}}=S_{\Gamma} ,$ $\left.T\right|_{\partial\Omega_{2}^{*}}=T_{\Gamma} ,$and Orlanski’s extrapolation is used at the outlet (Orlanski, 1976).

The conditions at the lower and upper boundaries consists of a condition along the co-normal to the bottom and a condition on the free air-sea surface:

$\begin{matrix} \left.\frac{\partial S}{\partial n}\right|_{\sigma=-1}=0 , & \left.\frac{\partial T}{\partial n}\right|_{\sigma=-1}=0 , \\ \left.\nu_{\rho}S_{\sigma}\right|_{\sigma=0}=0 , & \left.\nu_{\rho}T_{\sigma}\right|_{\sigma=0}=0 . \end{matrix}$ (11)

Finally, for the determination of $\nu$ the equation of kinetic energy of turbulence with a modified Montgomery’s formula for turbulence scales is used (Androsov and Voltzinger, 2005).

In the present investigation, this general numerical problem is solved on a uniform rectangular grid $Q_{\Delta}^{*}$ in the parallelepiped $Q^{*}$, that constitutes the mapping of a three-dimensional curvilinear grid $Q_{\Delta}$ generated in the physical domain $Q$. The grid has $33\times83\times40$ grid points. The time step was set to 60 s in order to ensure stability. The mesh size of the horizontal grid is varied in the range Δ_min_ = 50 m, Δ_max_ = 600 m. In the vertical, the equations are solved on 40 σ-levels.

At each time step the dynamics equations are solved in the following 4 steps.

1. as a first step, we compute a preliminary velocity field and the final temperature and salinity by semi-implicit advancement of the advective terms. The advection transport is determined by using a first, second, or third-order scheme in conjunction with a TVD procedure that controls the solution behavior in domains with steep gradients. Finally, we evaluate the water density and the baroclinic component of the pressure by integrating the density from the free surface to the sea-bottom.
2. In a second step, the full set of equations is integrated implicitly by splitting in the three coordinate directions.
3. In a third step, the 2-D elliptic equation for the determination of the sea level is implemented based on the vertically-averaged equations of continuity and velocity correction.
4. Finally, the 3-D velocity field is determined from the acquired knowledge of the sea level. The vertical contravariant components of the velocity are computed from the continuity equation, after which the Cartesian vertical velocity is obtained.

Overall, this scheme has a second-order accuracy for time and space variables. The main advantage of this method consists in the fact that no local smoothing or and filtration are needed in order to suppress the high-frequency spurious oscillations necessary.

**Supplementary Figures**

**
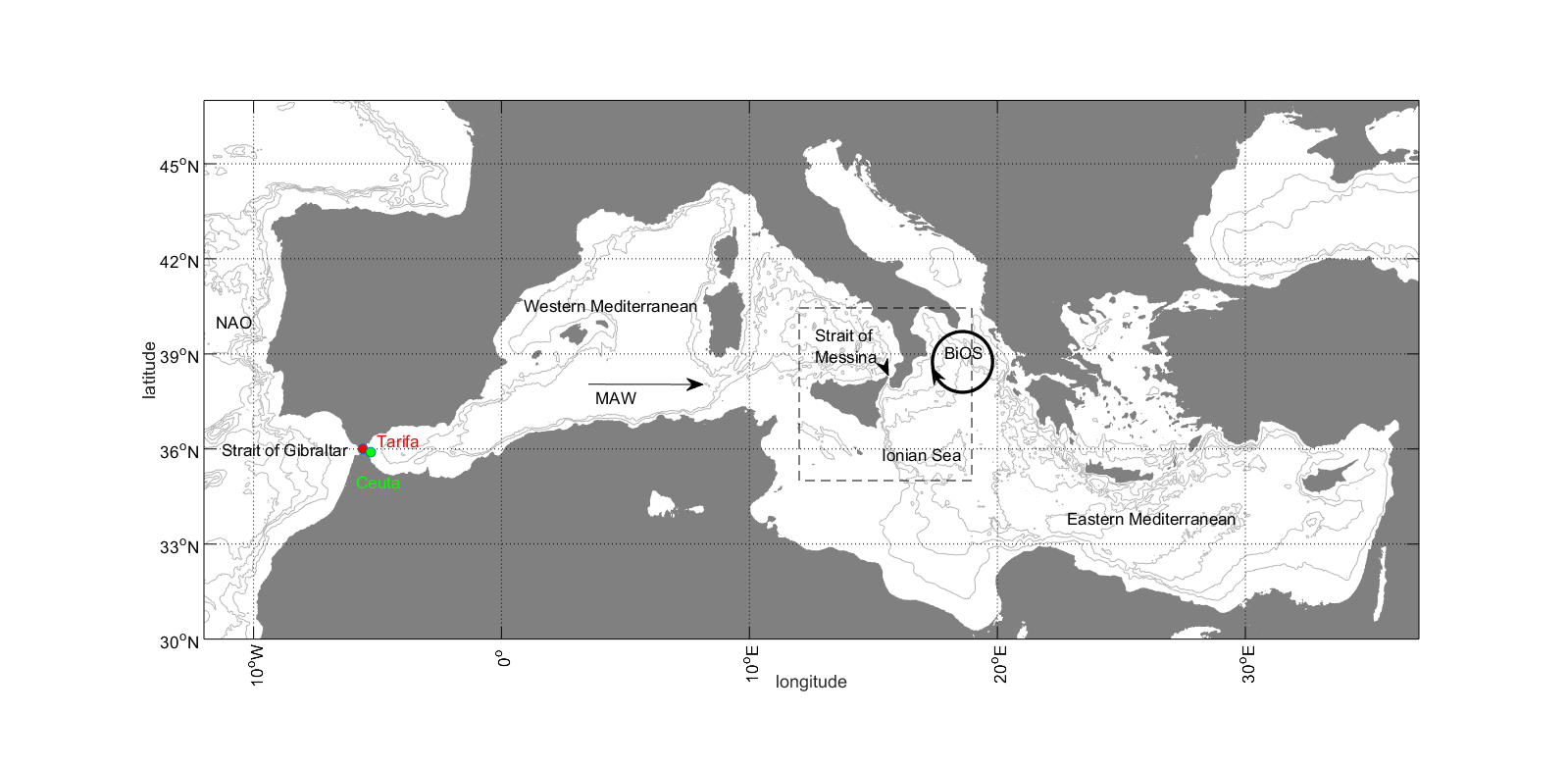
**

Supplementary Figure S1 – Map of the Mediterranean Sea with relevant geographic and oceanographic features. The dashed box indicates the study area illustrated in Fig. 2a.


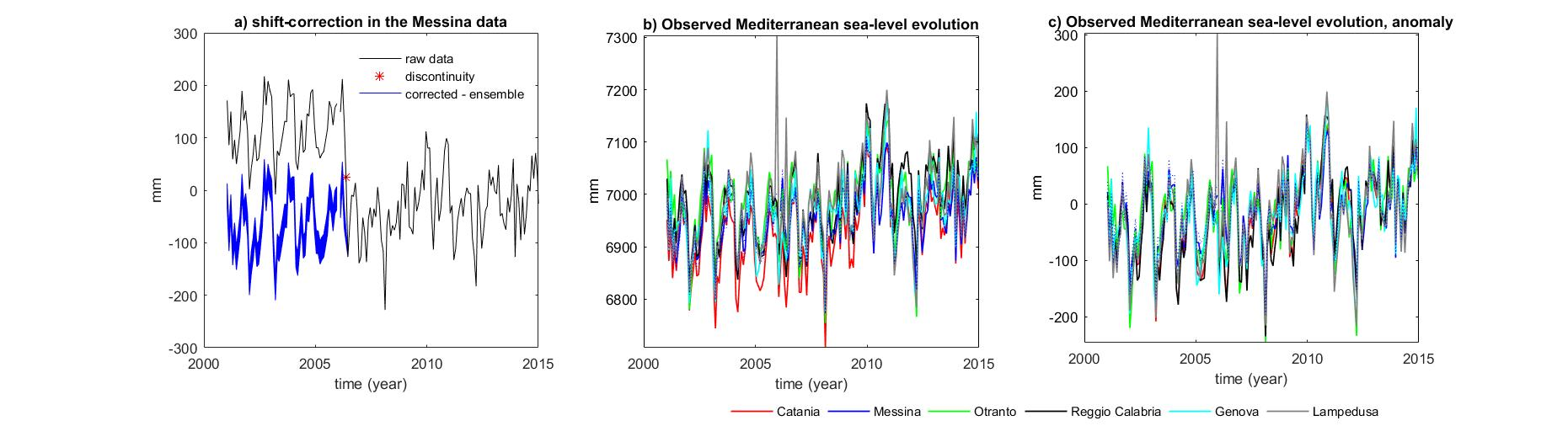


Supplementary Figure S2 – Preprocessing phases of the relative sea level data used in Figure 1a,b of the main text. (**a**) Results of the shift correction for the 2001-2015 SLH data of Messina as described in the “Data” section. (**b**) Raw monthly mean anomalies of SLH along the Italian coast, used in Figure 1a. A value of 7000 mm is added to the Messina data from panel **a** to align the series to the other ones. (**c**) Same as panel **b**, except data are anomalies from the 2007-2015 climatology of each series. Note that in the original series, the evolution is dominated by a marked annual seasonality. The temporary increase around 2010 is visually appreciable also in the raw data, but other features revealed in Figure 1a are not apparent.


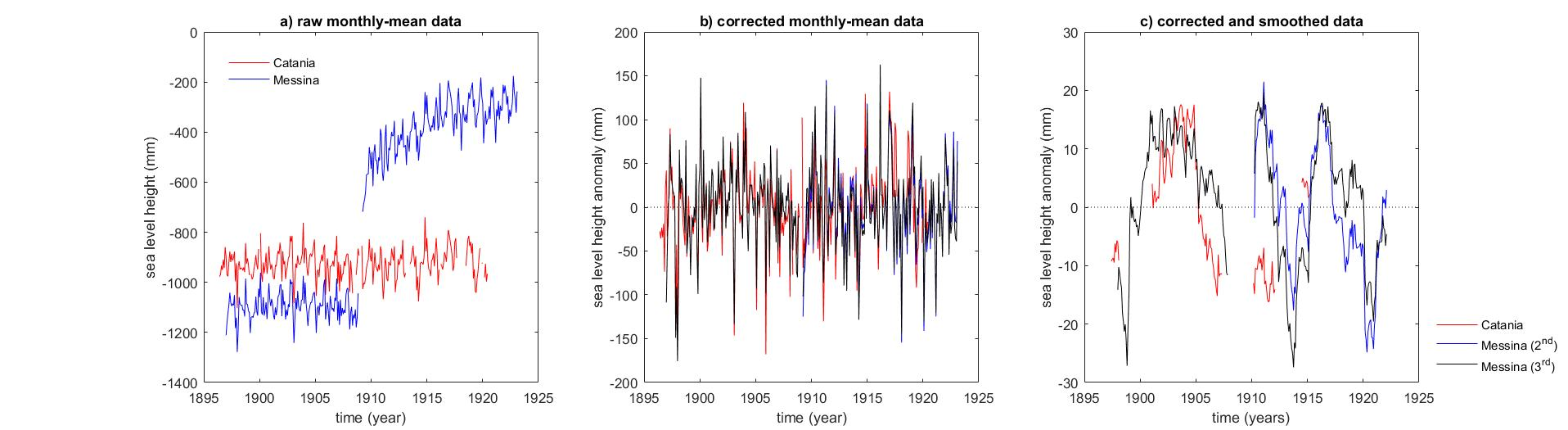


Supplementary Figure S3 - Preprocessing phases of the relative sea level data used in Figure 1c,d of the main text. (**a**) Raw monthly-mean data, showing the shift in the Messina record caused by the Dec. 28th 1908 earthquake. (**b**) Deseasonalized monthly-mean anomalies including shift correction for the Messina data based on two assumptions (2^nd^ and 3^rd^ order polynomial fit to the post-1908 data). (**c**) same as panel **b**, except data are smoothed with a 25-month running mean, as shown in Figure 1c. Note that in the original series, the evolution is dominated by a marked annual seasonality.

**References**

Androsov, A.A., Klevanny K.A., Salusti E.S. and Voltzinger N.E. (1995), Open boundary conditions for horizontal 2-D curvilinear-grid long-wave dynamics of a strait. Advances in Water Resources, 18, pp.267-276.

Androsov, A.A., Voltzinger N.E. (2005) The Straits of World ocean - the general approach to modeling. St. Petersburg: Nauka, 2005, 188 p., (in Russian).

Orlanski, I. (1976) A Simple Boundary Condition for Unbounded Hyperbolic Flows, J. Comput. Phys., Vol. 21, pp. 251-269.
